# Research on the Mechanism of Government–Industry–University–Institute Collaborative Innovation in Green Technology Based on Game–Based Cellular Automata

**DOI:** 10.3390/ijerph19053046

**Published:** 2022-03-05

**Authors:** Tuochen Li, Xinyu Zhou

**Affiliations:** School of Economics and Management, Harbin Engineering University, Harbin 150001, China; lituochen0409@163.com

**Keywords:** green technology innovation, government–industry–university–institute, collaborative innovation, dynamic evolution, evolutionary game theory, cellular automata

## Abstract

In order to ensure stable cooperation among the government, enterprise and university/institute in the green technology innovation process and guide an increasing number of innovation agents in the region to adopt cooperation, this paper studies the mechanism of green technology innovation. A tripartite evolutionary game model is established and the strategy choices of the government, industry and university/institute are analyzed through mathematical derivation. On this basis, the cellular automata theory is used to explore strategy choices of all innovation agents in the region from the perspective of a spatial game. From the numerical tests, the following results are obtained: increasing the cooperative innovation revenue, fairness of this revenue distribution or penalties for breach of contract can consolidate the cooperative relationship among the government, enterprise and university/institute, achieving the goal of guiding all innovation agents in the region to accept the collaborative innovation mode; regulating the government subsidy or government penalty can consolidate the cooperative relationship among participants in the pilot project, but cannot guide all innovation agents in the region accept the collaboration innovation mode. This paper’s results not only enrich the theory of government–industry–university–institute collaborative innovation in green technology, but provide ideas for stable cooperation mechanisms and comprehensive promotion of this collaborative innovation mode as well.

## 1. Introduction

An increasing number of countries actively formulate and implement green development plans, because limited resources and the pressure of the environment have been holding back their economic development [1,2,3]. Since green technology innovation can promote the transformation of industrial development modes and improve resource utilization efficiency, it has become the driving force for achieving green development. Enterprises, the principal party of green technology innovation, bear the responsibility of leading green development with technological innovation for a long time. On one hand, that green technology is conductive to the improvement of economic, environmental and social performance leads enterprises to take the initiative in carrying out green technology innovation [4,5]. On the other hand, enterprises have to adopt green technology innovation because of consumers’ green awareness increasing and the environmental regulation of the government becoming stricter [6]. It can be seen that green technology innovation is the development trend and essential way for enterprises.

Nowadays, green technology innovation modes can be divided into independent innovation and multi-agent collaborative innovation. Compared with independent innovation modes, the multi-agent collaborative innovation mode has the advantages of risk sharing, mutual benefit, win–win and sustainable innovation, which can significantly reduce the risk and threshold of green technology innovation projects that enterprises carry out [7]. Government–industry–university–institute collaborative innovation is a typical multi-agent collaborative innovation mode. Thereinto, the university/institute provides innovation impetus for enterprises by creating knowledge and cultivating talents; the enterprise alleviates investment pressure for the university/institute by financial support, and the government promotes sound progress in industry and maintains its reputation by guiding enterprise and university/institute cooperation. Therefore, this concept can be applied to green technology innovation as a relatively consummate innovation mode at the present stage [8,9].

The government–industry–university–institute collaborative innovation as a multi-agent system [10]; how to ensure stable cooperation among all participants is the primary problem which remains to be solved. On one hand, due to the complexity of green technology itself and the high risk involved in the process of green technology innovation, there are some barriers to collaborative innovation among the government, enterprise and university/institute, such as strategic emphasis shift [11], loss of competitiveness caused by core knowledge revealing [12], free-riding behavior [13] and illegal transfer of green technology patents [14]. On the other hand, that the government, enterprise and university/institute excessive pursue their own interests in maximizing benefits will shake the stability of green technology collaborative innovation, leading to a reduction in the quality of patent output and the patent enforcement rate of innovation system [15,16]. As a result, it is necessary to study how to strengthen the willingness of the government, enterprise and university/institute to cooperate and realize the maximization of common interests of the above innovation agents in the innovation system.

The fundamental purposes of green technology innovation are promoting the construction of ecological civilization and realizing the sustainable development of human society. It requires the joint efforts of the government, numerous enterprises and universities/institutes in the region. In the real society, the green technology innovation behavior complies with the diffusion effect [17], which represents that a successful/failed green technology collaborative innovation case has a positive/negative impact on the behaviors of innovation agents in the neighborhood. In addition, the existence of few enterprises with high independent innovation capacities and the government being profit-orientation lead to the mode of government–industry–university–institute collaborative innovation in green technology can only be guided rather than compelled [18,19]. Therefore, it is urgent to explore how to promote the mode of government–industry–university–institute collaborative innovation in green technology and guide an increasing number of innovation agents in the region to actively adopt cooperation strategy.

Collaborative innovation behavior in green technology innovation system embodies the process of a game among the government, industry, and university/institute. The behavior of each participant in the innovation system, to a certain extent, influences the decision making of other participants; meanwhile, it is also affected by their behaviors. In addition, that incomplete information in the process of green technology innovation causes each participant in the system to have to seek an optimal strategy through trial and error reflects the bounded rationality of individuals [20]. Therefore, the evolutionary game theory can be used to analyze the strategy selection and evolution regulation of the government, an enterprise and a university/institute in the process of innovation under a specific system. When studying green technology innovation behavior diffusion, the research emphasis changes from individual behavior to group behaviors. The government, enterprises and universities/institutes will compare their own profitability with the profitability of nearby same-type innovation agents, deciding whether they will adjust their current strategies or not (maintaining strategy or imitating the high-yield strategy [21]). Considering that the above process embodies the principle of a spatial game, the cellular automata model is introduced to analyze the diffusion path of green technology innovation behaviors in the region.

Based on the above, this paper studies the mechanism of government–industry–university–institute collaborative innovation in green technology through modeling, aiming to ensure stable cooperation among the government, enterprise and university/institute in the green technology innovation process, and guide an increasing number of innovation agents in the region to adopt a cooperative strategy. To capture the interactive behavior of the government, enterprise and university/institute in a specific project, a tripartite evolutionary game model is established for analyzing the evolutionary mechanism of green technology collaborative behavior in the innovation system. In order to study the diffusion path of green technology innovation behaviors, the cellular automata theory is used to explore strategy choices of all innovation agents in the region from the perspective of spatial game. First of all, this paper refers to the previous research studying the crucial factors that affect green technology collaborative behavior, and then extracts and defines the cost-benefit factors, equitable distribution factors, external incentive factors and penalty factors that are critical to green technology innovation. Secondly, by analyzing the game payoff matrix of green technology collaborative innovation strategies, the evolutionary game model for government–industry–university–institute collaborative innovation in green technology is established, and then the strategy choices of the government, industry and university/institute are analyzed through mathematical derivation. Meanwhile, the rule of cellular automata model is developed for describing the diffusion of the innovation behavior of all innovation agents in the region. Lastly, the influence of each factor on innovation agents’ green technology innovation behavior is simulated.

The remainder of this paper is structured as follows. Section 2 reviews the relevant literature on green technology innovation behavior among the government, enterprise and university/institute based on evolutionary game theory and cellular automata theory. Section 3 builds the evolutionary game model for green technology collaborative innovation behavior among the government, an enterprise and a university/institute, and the cellular automata model which is used to explore the diffusion of innovation behaviors of the government, enterprises and universities/institutes in the region. Section 4 represents the sensitivity analysis of different influence factors impacting on the evolutionary results. Section 5 offers conclusions and implications.

## 2. Literature Review

### 2.1. Green Technology and Green Technology Innovation

Green technology is the general term for a kind of technology which can improve the quality of ecological environment and promote the intensive use of energy resources [22]. Its concept was originally conceived for constructing a closed-loop industrial community without emissions; however, it finally developed into a realizable goal of reducing waste as a result of the above idea running counter to thermodynamics [23]. In essence, it is difficult to strictly discriminate green technology from grey technology because the green attributes of technology are identified on the basis of the degree of ecological influence. The key characteristic of green technology is that it attaches importance to environmental and social performance when pursuing economic performance promotion [24], and different types of green technologies have different innovation costs and risks [25].

Innovation is the primary driving force for development, and green technology innovation integrates environmental protection with economic development to fundamentally solve the problems of extensive development [26]. At present, a large number of scholars have discussed the importance of green technology innovation. Hong et al. [27] indicate that green technology innovation is conductive to improve resource efficiency and achieve win–win development of social energy conservation and emission reduction. Du et al. [28] point out that green technology innovation can enhance national or regional competitiveness. In this regard, Liu et al. [17] suggest strengthening input factors such as the government subsidy, private R&D funds and employment level in order to promote green technology innovation. Luo et al. [29] suggest building a scientific and reasonable indicator system and measurement method, making full use of input factors in order to improve green technology innovation output. Yu et al. [30] and Du et al. [31] propose to improve the innovation efficiency of green technology by optimizing the innovation mode.

In conclusion, with the increase in ecological problems, it is urgent for managers to use new theories and methods to examine the existing problems of green technology innovation. This is of great significance for increasing the output of green technology innovation and promoting the level of green development in a region or country.

### 2.2. Green Technology Collaborative Innovation Behavior among the Government, Enterprise and University/Institute

The government–industry–university–institute collaborative innovation as a benign mode of green technology innovation can effectively improve innovation system output. This is because enterprises are close to consumers, understanding market demand better than any other innovation agents and knowing which green technology innovation technologies have better market prospects [32]. Although universities/institutes do not have the ability to grasp market prospects, they have far more innovation ability than enterprises, which is conductive to the efficient implementation of green technology innovation projects [33]. As the main body of policy and regulation formulation, the government can adjust the incentive and supervision intensity of green technology innovation projects, creating a favorable external environment for enterprises and universities to cooperate [34,35]. However, in real society, innovation agents excessively pursuing their own interests in maximizing and free-riding behaviors cause the innovation system output to be lower than expected. As a result, scholars have studied the influence factors and process mechanism of green technology collaborative innovation behavior of the government, an enterprise and a university/institute based on the game theory. Based on a two-level low-carbon supply chain in the context of carbon trading, Zhang et al. [36] build a Stackelberg game model and point out that improving the level of subsides has a positive impact on the green technology innovation level of enterprises. Combined with the characteristics of the Chinese environmental regulation supervision system and evolutionary game theory, Deng et al. [37] indicate that increasing punishment for enterprises’ incomplete green technology innovation behavior is good for improving the probability of enterprises choosing the strategy of complete green technology innovation. Liu et al. [38] consider the cost and benefit of the government behavior and put forward that the probability of government regulation (supervision and innovation subsidies) decreases with the increase in regulatory costs and increases with the increase in regulatory benefits. Du et al. [31] think that green technology innovation requires multi-agents in the supply chain to cooperate, and analyze the impact of fairness concerns on the efficiency of green technology innovation through modeling.

To sum up, that previous research has excessively focused on the impacts of incentive factors and penalty factors on green technology innovation output causes the managers of modern organizations to lack an in-depth understanding of the decision-making process of innovation agents. Therefore, with the exception of the basic factor of cost–benefit, the fair distribution mechanism, incentive, and penalty mechanism should be analyzed so as to provide a theoretical basis for getting rid of the dilemma of government–industry–university–institute collaborative innovation.

Considering that the development of ecological civilization and sustainable development of human society depend on the joint efforts of the government, numerous enterprises and universities/institutes in the region, some scholars have studied the diffusion effect of green technology collaborative innovation behavior of innovation agents through empirical analysis. Marra et al. [39] analyze the green technology innovation behaviors of green technology companies in San Francisco, New York and London by using social networks, pointing out that the diffusion effect contributes to guide an increasing number of enterprises to formulate green technology innovation strategies and generate a spatial aggregation phenomenon. Huang et al. [40] construct a research framework based on the patent data of green transportation technologies and social network analysis methods, confirming that enterprises and universities/institutes will be influenced by successful cases and undertake collaborative innovation action actively. Liu et al. [21] analyze the spatial evolution characteristic of China’s green innovation from 2007 to 2017 by using social networks, indicating that geographical proximity intensifies the diffusion of green technology innovation behavior. Zhang et al. [41] simulate the effects of a carbon trading market, environmental taxes and innovation subsides on green technology diffusion in manufacturing firms in a BA scale-free network in China, summarizing the relationship between policy implementation and the diffusion effect.

Although the above studies have laid a foundation for green technology collaborative innovation, the following shortcomings exist: (1) due to the excessive focus on the posterior analysis of green technology innovation behavior and the lack of prediction research, there is no scientific basis for guiding more innovation agents in the region to choose collaborative innovation behavior; (2) that there are few studies on the spatial evolution of industry–university/institute collaborative innovation behavior has resulted in a lack of comprehensive understanding of the diffusion effect.

### 2.3. Applications of Evolutionary Game Theory and Cellular Automata

The development of evolutionary game theory stems from the biological theory of evolution. It uses the percentage of individuals who choose different pure strategies to replace the mixed strategies in game theory, and it can reflect the relationship between two or more individuals in real society [42]. Evolutionary game theory, as a mathematical theory of studying conflict or competition, focuses on how bounded rationality individuals optimize their benefits through adaptive learning over time in the process of repeated games. It has been widely used in production management, project management, supply chain management, social network and so on [43].

The evolutionary game theory is also applicable to research on government–industry–university–institute collaborative innovation in green technology. On the one hand, the purpose of research on green technology collaborative innovation is to clarify the decision-making paths of the above participants. The hypothesis of evolutionary game theory (participants exhibit bounded rationality) conforms to the basic characteristic of government–industry–university–institute collaborative innovation, and the evolutionary game model can reproduce the process in which each party adjusts its strategy on the basis of opponents’ behaviors until all participants in the innovation system are satisfied. On the other hand, the goal of research on green technology collaborative innovation is to explore the method of win–win cooperation among the government, enterprise and university/institute. Evolutionary game theory can present the relationship among the government, an enterprise and a university/institute in the form of algebraic formula and list feasible states and the prerequisites corresponding to them. It is convenient for managers to consolidate the cooperative relationship among green technology innovation participants through adjusting influence factors. Etzkowitz [44] establishes a framework of government–industry–university–institute collaborative innovation in green technology and theoretically discusses the game behavior among innovation agents in the process of innovation. Wang et al. [42] analyze the influence of consumers’ green consumption consciousness on the evolutionary path of industry–university/institute collaborative innovation by using evolutionary game theory. Hong et al. [27] prove the positive effect of green credit on the collaborative innovation behavior of green technology innovation agents by referring to evolutionary game theory.

The cellular automata model is a kind of discrete grid dynamic model; its time, space and state are all discrete, and spatial interaction and causality on time are completely specified in terms of a local relation [45]. It assumes that all individuals involved in the game are orderly located in a spatially directed graph and only play games with their nearest neighbors, and whether their strategies are maintained depends on the gains in-game. The cellular automata model is often used to simulate the spatio-temporal evolution of self-organizing systems.

Theoretically, the cellular automata model is applicable to analyze the diffusion of green technology innovation behavior as well. On one hand, each individual in real society is influenced by others belonging to its group and prefers to change its current state to improve their situation. On the other hand, previous studies have confirmed that there is spatial correlation in regional green development, and geographical proximity is able to intensify the diffusion of green technology innovation behavior [21,46]. Because the above evolution rule is consistent with the spatial game theory, the cellular automata model can be used to analyze the impact of the green technology innovation behaviors of the government, an enterprise and a university/institute in a pilot project in the region.

Accordingly, targeting the limitations of the existing research, a tripartite evolutionary game model for green technology innovation, which is guided by the government and involves the participation of enterprises and universities/institutes, is established, analyzing the evolutionary mechanism of innovation agents’ behaviors. On this basis, the cellular automata model is used to explore the diffusion path of green technology innovation behaviors of the government, enterprises and universities/institutes in the region. This paper mainly studies the following questions: (1) what factors have an impact on the evolution of green technology innovation? (2) how can these factors be controlled or adjusted to ensure stable cooperation among the government, an enterprise and a university/institute in the green technology innovation process? (3) how can these factors be controlled to guide an increasing number of innovation agents in the region to adopt cooperation strategies?

## 3. Model

### 3.1. Basic Description and Assumptions

The government, an enterprise and a university/institute are all important components of the green technology innovation mode. As the direct participants in green technology innovation, enterprises and universities/institutions coordinate their behaviors through changing revenue distribution, cost sharing and so on [47]. As the guide of green technology innovation, the government consolidates the cooperative relationship between the enterprise and the university/institute via laws, regulations and policies in the process of innovation [48]. To achieve sustainable development of human society through green technology innovation needs to go through two steps: establishment of a pilot project and promotion of the pilot project. Geographical proximity is conducive to the quick obtaining of information and the latest trends [49], theoretically, a successful/failed green technology collaborative innovation case will have a positive/negative impact on the behaviors of innovation agents in the neighborhood, and then promote/hinder collaborative innovation modes to expand in the region.

Therefore, this section builds the evolutionary game model and cellular automata model for government–industry–university–institute collaborative innovation in green technology. Based on the evolutionary game theory, the former integrates the influencing factors into the payoff function and constructs the game payoff matrix, and the strategy choices of the government, enterprise and university/institute under a specific system are analyzed through mathematical derivation. Based on the spatial game theory, innovation agents’ spatial location and information transmission mode are combined, formulating cellular automata rules in order to simulate the diffusion of green technology innovation behaviors. Thereinto, the government can adopt “participation” or “non-participation”, while enterprises and universities/institutes can adopt “cooperation” or “non-cooperation”. The objectives of this study are not only to enrich the theory of government–industry–university–institute collaborative innovation in green technology, but provide ideas for stable cooperation mechanisms and the comprehensive promotion of this collaboration innovation mode as well.

Our models are built upon a number of assumptions:

**Assumption** **1.** *The green technology innovation system is a uniformly mixed network, and one individual participant can play games with others in the innovation system*.

**Assumption** **2.** *The government, an enterprise and a university/institute in the green technology innovation system exhibit bounded rationality, and each participant adjusts its own strategy according to the others’ behaviors until evolutionary equilibrium*.

**Assumption** **3.** *The time, space and state of green technology innovation behavior of the government, numerous enterprises and universities/institutes in the region are all discrete, and heterogeneities among the same type of individuals can be ignored*.

### 3.2. The Evolutionary Game Model for Government–Industry–University–Institute Collaborative Innovation in Green Technology

#### 3.2.1. Model Variables and Game Payoff Matrix

Factors affecting participants green technology innovation behavior are as follows:

$V_{1}$, $V_{2}$, $V_{3}$: Initial cost of green technology innovation. The coefficients each corresponds to a participant (the government, enterprise and university/institute). Thereinto, enterprises and universities/institutes are the direct participants in green technology innovation, and the initial costs of green technology innovation are closely related to the type of green technology, their independent innovation abilities and so on. The government is the guide of green technology innovation, and its initial cost of green technology innovation is generated from supervision and innovation subsidies.

$R_{1}$, $R_{2}$: revenue of independent innovation. The coefficients each correspond to a participant (enterprise and university/institute), and $R_{1}$, $R_{2}>0$.

ΔR: revenue of cooperative innovation. Enterprises and universities/institutes complement each other’s advantages through cooperation, and then obtain additional revenue except for the revenue of independent innovation. Thereinto, ΔR>0.

α, β: represent the revenues of cooperative innovation of enterprise and university/institute accounting for percentage of the total revenue of cooperative innovation, respectively. Thereinto, α+β=1  (α, β>0).

$C_{1}$, $C_{2}$: maintenance cost of green technology collaborative innovation. The coefficients each corresponds to a participant (enterprise and university/institute). Cooperation is mutual, and one party alone adopting a cooperation strategy and investing in maintenance costs cannot achieve industry–university/institute collaborative innovation.

$I_{1}$, $I_{2}$: opportunity profit. The above coefficients present the profits which were obtained by the enterprise and university/institute, using maintenance costs to invest in other opportunities, respectively. Thereinto, $I_{1}$, $I_{2}>0$.

W: the penalty for breach of contract that an enterprise or university/institute has to suffer for betraying cooperation. It is agreed between the enterprise and university/institute, aiming to strengthen the cooperative relationship between them by raising the cost of a partner choosing free-riding behavior.

$t_{1}$, $t_{2}$: the above variables present the subsidy coefficients that the government renders for enterprises and universities/institutes adopting a cooperation strategy, respectively. The government subsidy embodies the incentive to green technology collaborative innovation, and its value reflects the level of the government subsidy.

$K_{1}$, $K_{2}$: government penalty. The above variables each corresponds to a participant (enterprise and university/institute) who chooses free-riding behavior. It reflects that the government exercises a supervision over the green technology collaborative innovation behaviors of enterprises and universities/institutes. Considering that an enterprise and a university/institute adopting non-cooperation at the same time are not encroaching upon their partner’s profit and the principle of government–industry–university–institute collaborative innovation is guiding but not compelling, the enterprise and university/institute will not be punished.

Q: supposing the revenue that the government adopts for participation is regarded as 0, Q(Q>0) would correspond to the loss that the government adopts for nonparticipation, including reputation loss caused by government’s omission, economic loss caused by green technology innovation hysteresis, and so on.

With the exception of the basic cost–benefit factors of the government, enterprise and university/institute (initial cost of green technology innovation V, revenue of independent innovation R, maintenance cost C, opportunity profit I and government loss Q), the cost–benefit variable (revenue of cooperative innovation ΔR), equitable distribution variable (proportions of cooperative innovation revenue distribution α and β), external incentive variable (government subsidy coefficient t) and penalty variables (penalty for breach of contract W and government penalty K) are systematically taken into account when the above factors were designed.

Using the above-mentioned factors, the game payoff matrices of the government–industry–university–institute collaborative innovation in green technology can be established as shown in Table 1 and Table 2.

The payoff functions of three participants in the game can be sorted into the following six situations:

Situation 1: when the government adopts a participation strategy and both the enterprise and university/institute adopt a cooperation strategy, the payoff of the enterprise is $R_{1}+\alpha \Delta R-\left(1-t_{1}\right)\left(C_{1}+V_{1}\right)$, which includes revenue of independent innovation $R_{1}$, revenue of cooperative innovation αΔR and cost of green technology innovation except for government subsidy $\left(1-t_{1}\right)\left(C_{1}+V_{1}\right)$. Similarly, the payoffs of university/institute and the government are $R_{2}+\beta \Delta R-\left(1-t_{2}\right)\left(C_{2}+V_{2}\right)$ and $-V_{3}-t_{1}\left(C_{1}+V_{1}\right)-t_{2}\left(C_{2}+V_{2}\right)$, respectively.

Situation 2: compared with situation 1, the payoff of the enterprise is $R_{1}+\alpha \Delta R-C_{1}-V_{1}$ because of a lack of government subsidy when the government adopts a nonparticipation strategy and both the enterprise and university/institute adopt a cooperation strategy. Similarly, the payoffs of the university/institute and the government are $R_{2}+\beta \Delta R-C_{2}-V_{2}$ and −Q, respectively.

Situation 3: when the government adopts a participation strategy, the enterprise adopts a cooperation strategy and the university/institute adopts a non-cooperation strategy, the payoff of the enterprise is $R_{1}-\left(1-t_{1}\right)\left(C_{1}+V_{1}\right)+W$, which includes revenue of independent innovation $R_{1}$, the cost of green technology innovation except for government subsidy $\left(1-t_{1}\right)\left(C_{1}+V_{1}\right)$ and the penalty for breach of contract W. The payoff of the university/institute is $R_{2}-V_{2}+I_{2}-W-K_{2}$, which includes revenue of independent innovation $R_{2}$, initial cost of green technology innovation $V_{2}$, opportunity profit $I_{2}$, penalty for breach of contract W and government penalty $K_{2}$. The payoff of the government is $-V_{3}-t_{1}\left(C_{1}+V_{1}\right)+K_{2}$, which includes the initial cost of green technology innovation $V_{3}$, government subsidy $t_{1}\left(C_{1}+V_{1}\right)$ and government penalty $K_{2}$. Similarly, when the government adopts a participation strategy, the enterprise adopts a non-cooperation strategy and the university/institute adopts a cooperation strategy, the payoffs of the enterprise, university/institute and the government are $R_{1}-V_{1}+I_{1}-W-K_{1}$, $R_{2}-\left(1-t_{2}\right)\left(C_{2}+V_{2}\right)+W$ and $-V_{3}-t_{2}\left(C_{2}+V_{2}\right)+K_{1}$, respectively.

Situation 4: compared with situation 3, due to a lack of government subsidy, the payoffs of the enterprise, university/institute and the government are $R_{1}-C_{1}-V_{1}+W$, $R_{2}-V_{2}+I_{2}-W$ and −Q, respectively, when the government adopts nonparticipation strategy, the enterprise adopts cooperation strategy and the university/institute adopts non-cooperation strategy. Similarly, when the government adopts a nonparticipation strategy, the enterprise adopts a non-cooperation strategy and the university/institute adopts a cooperation strategy, the payoffs of enterprise, university/institute and the government are $R_{1}-V_{1}+I_{1}-W$, $R_{2}-C_{2}-V_{2}+W$ and −Q, respectively.

Situation 5: when the government adopts a participation strategy and neither the enterprise nor the university/institute adopt a cooperation strategy, the payoff of the enterprise is $R_{1}-V_{1}+I_{1}$, which includes revenue of independent innovation $R_{1}$, initial cost of green technology innovation $V_{1}$ and opportunity profit $I_{1}$. Similarly, the payoffs of the university/institute and the government are $R_{2}-V_{2}+I_{2}$ and $-V_{3}$, respectively.

Situation 6: compared with situation 5, the revenues of the enterprise and university/institute do not change, and the payoff of the government is $-V_{3}$ when the government adopts a nonparticipation strategy and neither the enterprise nor the university/institute adopt a cooperation strategy.

#### 3.2.2. Dynamic Evolutionary Equilibrium and Stability

The government, enterprise and university/institute in the green technology innovation system exhibit bounded rationality, and one game adjusts its strategy on the basis of others’ behaviors until equilibrium is reached. Based on Table 1 and Table 2, assume the proportion of adopting a cooperation strategy for the enterprise is x, and the proportion of adopting a non-cooperation strategy is (1−x). Similarly, assume the proportion of adopting a cooperation strategy for the university/institute is y, and the proportion of adopting a non-cooperation strategy is (1−y), assuming the proportion of adopting a participation strategy for the government is z, and the proportion of adopting a nonparticipation strategy is (1−z). The replicator dynamics equation is used to solve the approximate solution of evolutionary equilibrium.

When an enterprise chooses a cooperation strategy, its average revenue is defined in Equation (1):

(1) $$U_{1}=yz\left[R_{1}+\alpha \Delta R-\left(1-t_{1}\right)\left(C_{1}+V_{1}\right)\right]+\left(1-y\right)z\left[R_{1}-\left(1-t_{1}\right)\left(C_{1}+V_{1}\right)+W\right]\;\;+y\left(1-z\right)\left(R_{1}+\alpha \Delta R-C_{1}-V_{1}\right)+\left(1-y\right)\left(1-z\right)\left(R_{1}-C_{1}-V_{1}+W\right)$$

The average revenue of an enterprise choosing a non-cooperation strategy is defined in Equation (2):

(2) $$U_{1}^{\prime }=yz\left[R_{1}-V_{1}+I_{1}-W-K_{1}\right]+\left(1-y\right)z\left[R_{1}-V_{1}+I_{1}\right]+y\left(1-z\right)\left(R_{1}-V_{1}+I_{1}-W\right)\;\;+\left(1-y\right)\left(1-z\right)\left(R_{1}-V_{1}+I_{1}\right)$$

Combining the above Equations (1) and (2), the average total revenue of the enterprise is:

(3) $${\bar{U}}_{1}=xU_{1}+\left(1-x\right)U_{1}^{\prime }$$

when a university/institute chooses a cooperation strategy, its average revenue is defined in Equation (4):

(4) $$U_{2}=xz\left[R_{2}+\beta \Delta R-\left(1-t_{2}\right)\left(C_{2}+V_{2}\right)\right]+\left(1-x\right)z\left[R_{2}-\left(1-t_{2}\right)\left(C_{2}+V_{2}\right)+W\right]\;\;+x\left(1-z\right)\left(R_{2}+\beta \Delta R-C_{2}-V_{2}\right)+\left(1-x\right)\left(1-z\right)\left(R_{2}-C_{2}-V_{2}+W\right)$$

The average revenue of a university/institute choosing a non-cooperation strategy is defined in Equation (5):

(5) $$U_{2}^{\prime }=xz\left(R_{2}-V_{2}+I_{2}-W-K_{2}\right)+\left(1-x\right)z\left(R_{2}-V_{2}+I_{2}\right)+x\left(1-z\right)\left(R_{2}-V_{2}+I_{2}-W\right)\;\;+\left(1-x\right)\left(1-z\right)\left(R_{2}-V_{2}+I_{2}\right)$$

Combining the above Equations (4) and (5), the average total revenue of the university/institute is:

(6) $${\bar{U}}_{2}=yU_{2}+\left(1-y\right)U_{2}^{\prime }$$

when the government chooses a participation strategy, its average revenue is defined in Equation (7):

(7) $$U_{3}=xy\left[-V_{3}-t_{1}\left(C_{1}+V_{1}\right)-t_{2}\left(C_{2}+V_{2}\right)\right]+x\left(1-y\right)\left[-V_{3}-t_{1}\left(C_{1}+V_{1}\right)+K_{2}\right]\;+\left(1-x\right)y\left[-V_{3}-t_{2}\left(C_{2}+V_{2}\right)+K_{1}\right]+\left(1-x\right)\left(1-y\right)\left(-V_{3}\right)$$

The average revenue of the government choosing a nonparticipation strategy is defined in Equation (8):

(8) $$U_{3}^{\prime }=-xyQ-x\left(1-y\right)Q-\left(1-x\right)yQ-\left(1-x\right)\left(1-y\right)Q$$

Combining the above two Equations (7) and (8), the average total revenue of the government is:

(9) $${\bar{U}}_{3}=zU_{3}+\left(1-z\right)U_{3}^{\prime }$$

The replicator dynamics equation of the enterprise is:

(10) $$F\left(x\right)=\frac{dx}{dt}=x\left(U_{1}-{\bar{U}}_{1}\right)=x\left(1-x\right)\left[y\left(zK_{1}+\alpha \Delta R\right)+zt_{1}\left(C_{1}+V_{1}\right)-C_{1}+W-I_{1}\right]$$

The replicator dynamics equation of the university/institute is:

(11) $$F\left(y\right)=\frac{dy}{dt}=y\left(U_{2}-{\bar{U}}_{2}\right)=y\left(1-y\right)\left[x\left(zK_{2}+\beta \Delta R\right)+zt_{2}\left(C_{2}+V_{2}\right)-C_{2}+W-I_{2}\right]$$

The replicator dynamics equation of the government is:

(12) $$F\left(z\right)=\frac{dz}{dt}=z\left(U_{3}-{\bar{U}}_{3}\right)=z\left(1-z\right)\left\{-x\left[t_{1}\left(C_{1}+V_{1}\right)-K_{2}\right]-y\left[t_{2}\left(C_{2}+V_{2}\right)-K_{1}\right]-xy\left(K_{1}+K_{2}\right)-V_{3}+Q\right\}$$

In an evolutionary game model, the trajectory emitted from an arbitrarily small neighborhood evolves towards a certain asymptotically stable balance point, which is called ESS [50]. If a sufficient proportion of participants adopt a certain strategy that achieves ESS, then the system will remain stable. According to the ESS definition, assume F(x)=0, F(y)=0 and F(z)=0, then eight possible equilibrium points are obtained: $E_{1}=\left(0,0,0\right)$, $E_{2}=\left(0,0,1\right)$, $E_{3}=\left(0,1,0\right)$, $E_{4}=\left(1,0,0\right)$, $E_{5}=\left(0,1,1\right)$, $E_{6}=\left(1,0,1\right)$, $E_{7}=\left(1,1,0\right)$ and $E_{8}=\left(1,1,1\right)$.

Friedman’s study [51] provided the ESS condition for the evolutionary game. Specifically, any state that satisfies all eigenvalues of the Jacobian matrix are non-positive is ESS. Using replicator dynamics in Equations (10)–(12), Jacobian matrix J can be expressed as:

(13) $$J=\left[\begin{array}{ccc} \left(1-2x\right)\left[y\left(zK_{1}+\alpha \Delta R\right)+zt_{1}\left(C_{1}+V_{1}\right)-C_{1}+W-I_{1}\right] & x\left(1-x\right)\left(zK_{1}+\alpha \Delta R\right) & x\left(1-x\right)\left[yK_{1}+t_{1}\left(C_{1}+V_{1}\right)\right]\\ y\left(1-y\right)\left(zK_{2}+\beta \Delta R\right) & \left(1-2y\right)\left[x\left(zK_{2}+\beta \Delta R\right)+zt_{2}\left(C_{2}+V_{2}\right)-C_{2}+W-I_{2}\right] & y\left(1-y\right)\left[xK_{2}+t_{2}\left(C_{2}+V_{2}\right)\right]\\ z\left(1-z\right)\left[-t_{1}\left(C_{1}+V_{1}\right)+K_{2}-y\left(K_{1}+K_{2}\right)\right] & z\left(1-z\right)\left[-t_{2}\left(C_{2}+V_{2}\right)+K_{1}-x\left(K_{1}+K_{2}\right)\right] & \left(1-2z\right)\left\{\begin{array}{c} -x\left[t_{1}\left(C_{1}+V_{1}\right)-K_{2}\right]-y\left[t_{2}\left(C_{2}+V_{2}\right)-K_{1}\right]\\ -xy\left(K_{1}+K_{2}\right)-V_{3}+Q \end{array}\right\} \end{array}\right]$$

The eigenvalues of Jacobian matrix J are obtained through calculation as shown in Table 3. Given that government–industry–university–institute collaborative innovation in green technology is complementary, and analysis of the sign of eigenvalue corresponding to different equilibrium points without losing generality is convenient, this paper assumes that the total net profits of an enterprise, a university/institute and the government when they choose collaborative innovation are more that the total net profits of them when each does things in its own way, that is, $-V_{3}-t_{1}\left(C_{1}+V_{1}\right)-t_{2}\left(C_{2}+V_{2}\right)+Q>0$, $\alpha \Delta R-C_{1}-I_{1}+W>0$ and $\beta \Delta R-C_{2}-I_{2}+W>0$. The local stability of equilibrium for the three situations is shown in Table 4. Scenario 1: if $t_{1}\left(C_{1}+V_{1}\right)-C_{1}+W-I_{1}<0$ and $t_{2}\left(C_{2}+V_{2}\right)-C_{2}+W-I_{2}<0$, $E_{2}\left(0,\;0,\;1\right)$ and $E_{8}\left(1,\;1,\;1\right)$ are ESS—that is, enterprise and university/institute adopt non-cooperation strategy and the government adopts participation strategy, or all of them choose collaborative innovation. Scenario 2: if $-C_{1}+W-I_{1}>0$ or $-C_{2}+W-I_{2}>0$, $E_{8}\left(1,\;1,\;1\right)$ is ESS, which corresponds to the case that the enterprise and the university/institute adopt a cooperation strategy and the government adopts a participation strategy. Scenario 3: if $t_{1}\left(C_{1}+V_{1}\right)-C_{1}+W-I_{1}>0$ and $-C_{1}+W-I_{1}<0$, or $t_{2}\left(C_{2}+V_{2}\right)-C_{2}+W-I_{2}>0$ and $-C_{2}+W-I_{2}<0$, $E_{8}\left(1,\;1,\;1\right)$ is ESS, which corresponds to the case that the enterprise and the university/institute adopt a cooperation strategy and the government adopts a participation strategy.

### 3.3. The Cellular Automata Model for Government–Industry–University–Institute Collaborative Innovation in Green Technology

In order to construct the cellular automata for government–industry–university–institute collaborative innovation in green technology to simulate green technology innovation behavior diffusion in the region, the basic assumptions and payoff matrices in Section 3.2.1 are applied in this section, and its basic components (cell (d), cell space ($L^{2}$), neighbour (N), cell state (S) and evolution rule (rule)) are clarified [52,53,54]. The specific settings are as follows:

In terms of cell and cell state setting, assume d = {enterprise, university/institute, the government}, that is, each grid in cell space contains three cells: an enterprise, a university/institute and the government. For enterprise cells, their cell state is $S_{1}\;$= {cooperation, non-cooperation}; for university/institute cells, their cell state is $S_{2}\;$= {cooperation, non-cooperation}; for the government cells, their cell state is $S_{3}\;$= {participation, nonparticipation}.

In terms of cell space and neighbor setting, this paper uses two-dimensional cell space to simulate the diffusion of green technology innovation behavior and assumes that the cell space is a square grid with side length L = 100 and with periodic boundary conditions. Meanwhile, assume that the cellular automata are made up of the Moore neighbor type.

In terms of the evolution rule, assume the cell state at T+1 is affected by its own state and the state which corresponds to one of its neighbors randomly selected at T. This can be expressed as:

(14) $$S_{r}^{T+1}=rule\left(S_{r}^{T},S_{rN}^{T+1}\right)$$

Thereinto, $S_{r}^{T+1}$ and $S_{r}^{T}$ represent the states of cells located in r at T+1 and T, respectively. $S_{rN}^{T}$ represents the state of cell N, one of the neighbors of the cells located in r randomly selected for referring, at T. The evolution rule at each moment is described as follows:

(1) When T=0, assume that cooperative enterprise cells account for a of the total of all enterprise cells, the proportion of non-cooperative enterprise cells is (1−a) and they are randomly distributed in grids of the cell space. At the same time, assume that cooperative university/institute cells account for b of the total of all university/institute cells, the proportion of non-cooperative university/institute cells is (1−b) and they are randomly distributed in grids of the cell space. Assume that the government cells corresponding to the participation strategy account for c of the total of all the government cells, the proportion of the government cells corresponding to nonparticipation strategy is (1−c) and that they are randomly distributed in grids of the cell space.
(2) When T=1, the three cells located in r will confirm to each other’s states and their own payoffs at this time. Define the payoff of the cell located in r at this moment as $\pi \left(S_{r}^{1}\right)$. Meanwhile, cells located in r will randomly select a cell with the same type from their neighbors for comparison on payoff. Define the payoff of the neighbor cell at T=1 as $\pi \left(S_{rN}^{1}\right)$. If $\pi \left(S_{r}^{1}\right)\geq \pi \left(S_{rN}^{1}\right)$, the cells located in r will maintain their current states next time; if $\pi \left(S_{r}^{1}\right)<\pi \left(S_{rN}^{1}\right)$, they will imitate the states of their neighbors to choose $S_{rN}^{1}$.
(3) When T=2, the three cells located in r will convert their state from $S_{r}^{1}$ to $S_{r}^{2}$ according to the result of comparison on payoff in the previous stage, selecting their neighbors randomly again to compare the payoff at this time. This process is repeated until the iteration number ends.

## 4. Simulation and Discussion

In order to clearly and intuitively describe the dynamic evolution behaviors of the government, enterprise and university/institute in the process of green technology innovation, a numerical simulation analysis was used to discuss the influences of the values of the cost–benefit variable, equitable distribution variable, external incentive variable and penalty variables on the stability of the evolutionary game model and the regional promotion of the government–industry–university–institute collaborative innovation mode. Due to the diversity of the research data and the abstractness of the hypothetical variables, it is complicated to set up the initial value of the model by an actual assignment method based on realistic data. This paper refers to related works [55,56] and adopts the equilibrium assignment method (subjective assignment). The specific setting is shown in Table 5. Although this assignment method is not supported by historical data, it can be used to analyze and predict the evolution trend of government–university–industry collaborative innovation, and compare the effects of changes before and after the coefficients [57]. In addition, the initial participation intention of the government and initial cooperation intentions of enterprise and university/institution in the innovation system are all set at 0.3.

### 4.1. Influence of Revenue of Cooperative Innovation ΔR

**Figure 1:**
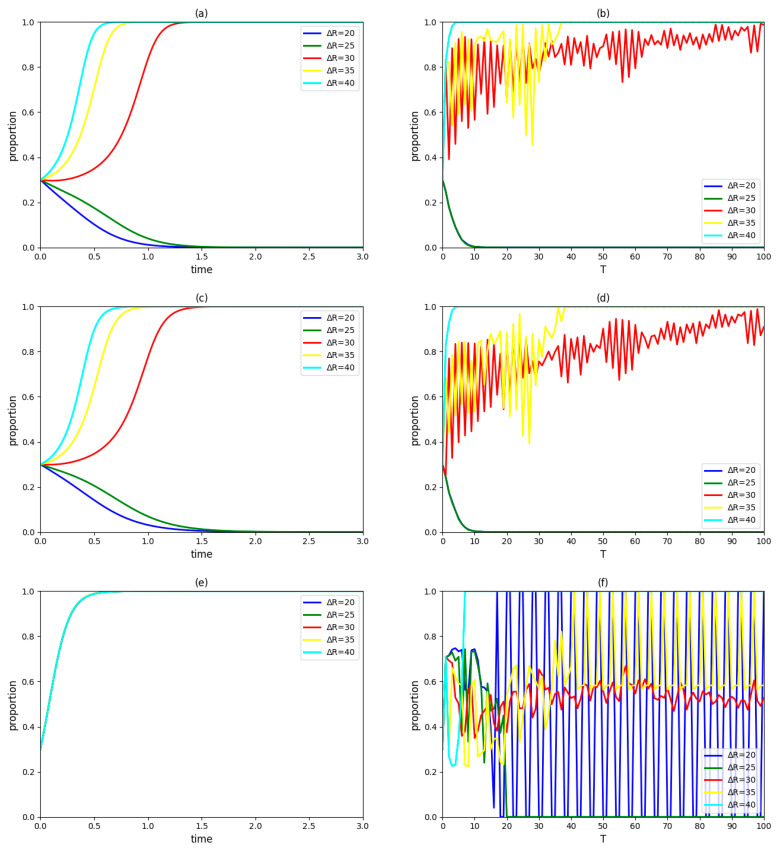
(**a**) The evolutionary paths of enterprise strategies with different cooperative innovation revenues. (**b**) The proportion of cooperative enterprises in the region over time. (**c**) The evolutionary paths of university/institute strategies with different cooperative innovation revenues. (**d**) The proportion of cooperative universities/institutes in the region over time. (**e**) The evolutionary paths of the government strategies with different cooperative innovation revenues. (**f**) The implementation of the proportion of the government adopting a participation strategy in the region over time.

Assume ΔR takes 20, 25, 30, 35 and 40, respectively, and keep other variables unchanged. The dynamic evolution process of strategic choices of the government, an enterprise and a university in the innovation system and the proportions of those three types of innovation agents accepting the collaboration innovation mode in the region over time are observed as Figure 1.

According to Figure 1a,c,e, when the revenue of cooperative innovation ΔR decreases to 20 or 25, both enterprise and university/institute in the innovation system will eventually adopt a non-cooperation strategy; furthermore, they will reach a stable state faster under the former condition. When the revenue of cooperative innovation ΔR increases to 35 or 40, both enterprise and university/institute in the innovation system will eventually adopt a cooperation strategy; furthermore, they will reach a stable state faster under the latter condition. The government’s final decision and the rate at which a stable state is achieved are not affected by the revenue of cooperative innovation ΔR. In terms of the collaborative innovation mode of green technology with government participation and cooperation between an enterprise and a university/institute, changing cooperative innovation revenue directly affects the profits of the enterprise and the university/institute, and then has an impact on their cooperation intentions [58,59]; however, it will not affect the government’s profit. Therefore, under the premise of the government being inclined to participate in green technology innovation, increasing cooperative innovation revenue is conductive to promoting the building of the pilot of government–industry–university–institute collaborative innovation; however, reducing this revenue hinders completion of the pilot.

Figure 1b,d,f show that cooperation is the mainstream strategy of enterprises and universities/institutes when Δ*R* = 30; however, it cannot be thoroughly implemented in the region because the proportions of groups of cooperative enterprises and cooperative universities/institutes will gradually increase and finally remain high and volatile. When ΔR decreases to 20 or 25, the proportions of groups of cooperative enterprises and cooperative universities/institutes will gradually decrease to extinction with the increasing iteration number. Moreover, there is little difference between the rates of enterprises or universities/institutes at which a stable state is achieved under two conditions. When ΔR increases to 35 or 40, the proportions of groups of enterprises and universities/institutes will gradually increase with the increasing iteration number. Moreover, both enterprises and universities/institutes will research a stable state faster under the latter condition. It can be concluded that increasing the cooperative innovation revenue in a pilot project has a positive impact on collaborative behaviors of neighboring innovation agents (enterprises and universities/institutes), and the greater the increase in this revenue, the easier the industry–university/institute collaborative innovation will be accepted in the region. There is a complex relationship between the revenue of cooperative innovation and the government’s strategic choice. When ΔR = 20, 25, 30 and 35, it is difficult for the government to take participating in industry–university/institute collaborative innovation as a macrostrategy to be fully implemented in the region, which is manifested by its weak and unstable willingness to cooperate. When ΔR = 40, participation strategy can be used as the government’s macrostrategy to enable an overall layout in the region.

In general, increasing the revenue of cooperative innovation, to a certain extent, can stimulate innovation agents’ willingness to cooperate; however, this measure ensuring stable cooperation among the government, enterprise and university/institute in the pilot project will not necessarily lead to the comprehensive promotion of the government–industry–university–institute collaborative innovation mode. Therefore, in order to ensure that all innovation agents in the region actively adopt the green technology collaborative innovation mode, a great demand is placed on the increase in cooperative innovation revenue.

### 4.2. Influence of Proportions of Cooperative Innovation Revenue Distribution α and β

**Figure 2:**
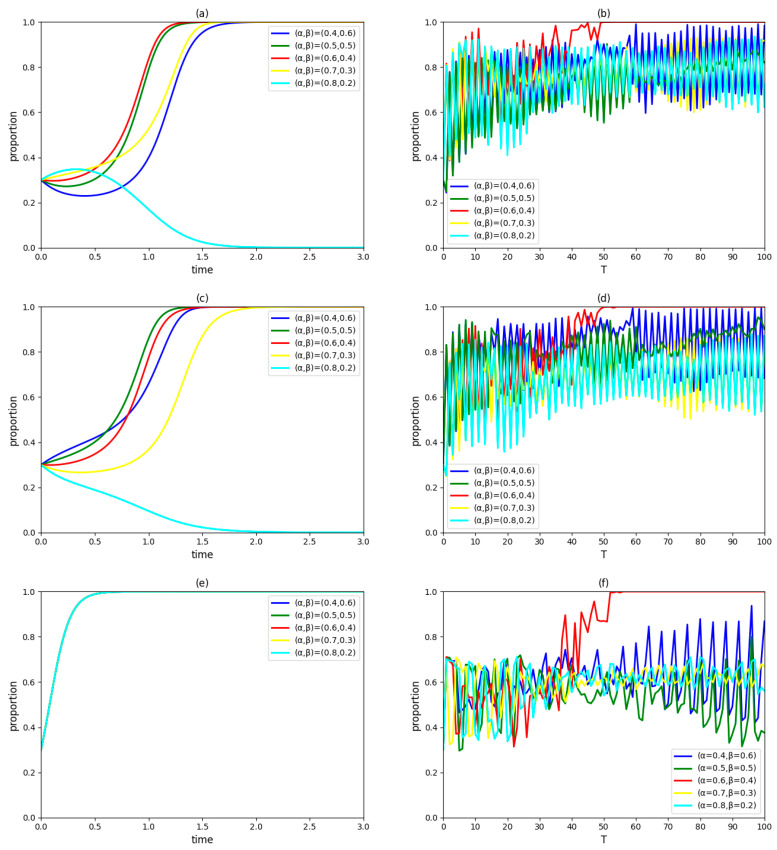
(**a**) The evolutionary paths of enterprise strategies with different proportions of cooperative innovation revenue distribution. (**b**) The proportion of cooperative enterprises in the region over time. (**c**) The evolutionary paths of university/institute strategies with different proportions of cooperative innovation revenue distribution. (**d**) The proportion of cooperative universities/institutes in the region over time. (**e**) The evolutionary paths of government strategies with different proportions of cooperative innovation revenue distribution. (**f**) The implementation of the proportion of the government adopting a participation strategy in the region over time.

Assume (α, β) takes (0.4, 0.6), (0.5, 0.5), (0.6, 0.4), (0.7, 0.3) and (0.8, 0.2), respectively, and keep other variables unchanged. The dynamic evolution process of the strategic choices of the government, an enterprise and a university in the innovation system and the proportions of those three types of innovation agents accepting the collaboration innovation mode in the region over time are observed as Figure 2.

In Figure 2a,c,e, when (α, β)=(0.6, 0.4), the probabilities of enterprises and universities/institutes in the innovation system adopting a cooperation strategy will continue to increase, which shows that this distribution of cooperative innovation revenue is fair and their cooperation targets are clear [43]. When (α, β)=(0.4, 0.6), (0.5, 0.5) or (0.7, 0.3), the proportions of enterprises and universities/institutes in the innovation system adopting a cooperation strategy will increase first and then decrease, which indicates that, although the revenues of cooperative innovation obtained by enterprises and universities/institutes are within their acceptable ranges, respectively, the unfair distribution of cooperative innovation revenue makes them give priority to non-cooperation. When (α, β)=(0.8, 0.2), the revenue of cooperative innovation obtained by the university/institute is lower than its acceptable range, which indirectly causes the enterprise to withdraw from cooperation. Since cooperative innovation revenue does not affect the government’s willingness to cooperate, the government final decision and its rate at which a stable state is achieved are not affected by the proportions of this revenue distribution.

According to Figure 2b,d,f, when (α, β)=(0.6, 0.4), all enterprises and universities/institutes will eventually adopt cooperation strategy, and participating in industry-university/institute collaborative innovation will become the strategy of the government to be fully implemented in the region. However, other cooperative innovation revenue distribution schemes mentioned in the study cannot achieve the above goal, which are manifested by continuous fluctuation in the proportion of innovation agents accepting the collaborative innovation mode. Combined with Figure 2a,c,e, it can be seen that the absolute fairness of cooperative innovation revenue distribution in a pilot project has a positive impact on the collaborative behaviors of neighboring innovation agents, which ensures the government–industry–university–institute collaborative innovation mode diffused with smooth progress in the region.

Proportions of cooperative innovation revenue distribution α and β reflect the fairness of benefit distribution in the process of green technology innovation. The higher the fairness of benefit distribution is, the stronger the cooperative willingnesses of the government, enterprise and university/institute will be. However, this measure ensuring stable cooperation among the government, enterprise and university/institute in the pilot project will not necessarily lead to the comprehensive promotion of the government–industry–university–institute collaborative innovation mode. Therefore, in order to ensure that all innovation agents in the region actively adopt green technology collaborative innovation mode, a great demand is being placed on the fairness of cooperative innovation revenue distribution.

### 4.3. Influence of Government Subsidy Coefficients $t_{1}$ and $t_{2}$

**Figure 3:**
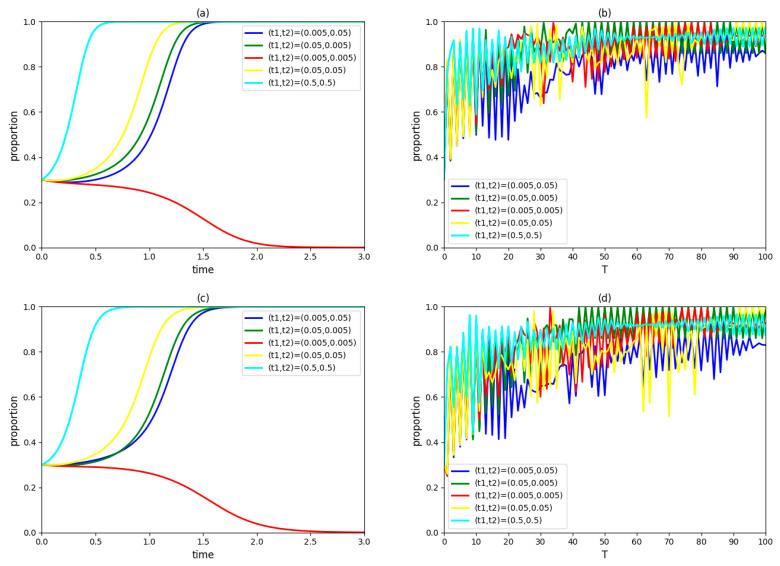

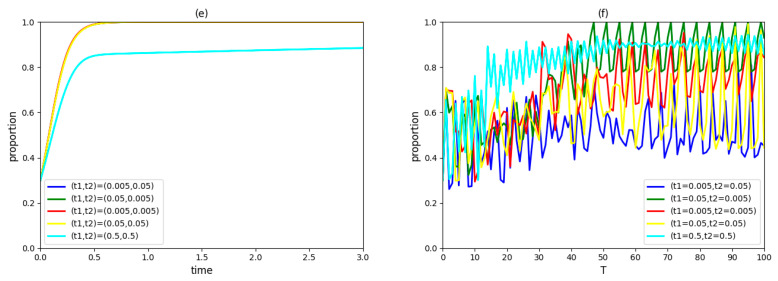
(**a**) The evolutionary paths of enterprise strategies with different government subsidy coefficients. (**b**) The proportion of cooperative enterprises in the region over time. (**c**) The evolutionary paths of university/institute strategies with different government subsidy coefficients. (**d**) The proportion of cooperative universities/institutes in the region over time. (**e**) The evolutionary paths of government strategies with different government subsidy coefficients. (**f**) The implementation of the proportion of the government adopting a participation strategy in the region over time.

Assume $\left(t_{1},\;t_{2}\right)$ takes (0.005, 0.05), (0.05, 0.005), (0.005, 0.005), (0.05, 0.05) and (0.5,0.5), respectively, and keep other variables unchanged. The dynamic evolution process of strategic choices of the government, an enterprise and a university in the innovation system and the proportions of those three types of innovation agents accepting the collaboration innovation mode in the region over time are observed as Figure 3.

Figure 3a,c,e show that enterprises and universities/institutes in the innovation system adopt a cooperation strategy when $\left(t_{1},\;t_{2}\right)=\left(0.005,\;0.005\right)$. When $\left(t_{1},\;t_{2}\right)=\left(0.005,\;0.05\right)$, (0.05, 0.005), (0.05, 0.05) or (0.5, 0.5), enterprises and universities/institutes in the innovation system will eventually adopt a cooperation strategy; furthermore, both of them will research a stable state faster under the latter condition. In terms of the collaborative innovation mode of green technology with government participation and cooperation between an enterprise and a university/institute, changing government subsidies directly affect the profits of the enterprise and the university/institute, which then has an impact on their cooperation intentions [60]. Therefore, increasing government subsidies can encourage the enterprise and the university/institute to adopt a cooperation strategy. However, increasing government subsidies is not unbridled. In the government subsidies schemes settled, the willingness of the government to participate in green technology innovation decreases significantly when both $t_{1}$ and $t_{2}$ increase to 0.5. This is because extremely high government subsidies will reduce the government’s profit and then weaken the government’s willingness to participate in green technology innovation.

In Figure 3b,d,f, the proportions of groups of the government, enterprise and university/institute accepting the collaborative innovation mode are unlikely to achieve 100%; moreover, proportions of the same innovation agents fluctuate within a similar range under different government subsidy coefficients. It can be inferred that regulating and controlling government subsidies not only has difficulty in leading to all enterprises and universities/institutes adopting a cooperation strategy (even the government cannot actively participating in green technology collaborative innovation), but also does not significantly promote or inhibit the cooperative innovation behaviors of innovation agents in the region. The reason for this phenomenon is that increasing government subsidies in a pilot project has a positive impact on the collaborative behaviors of neighboring innovation agents (enterprises and universities/institutes), but a negative impact on the government’s participation behavior. Therefore, it is hard to make all innovation agents in the region work towards the same goal merely through government subsidies.

Government subsidy is a common incentive method, and the subsidy coefficient reflects the incentive intensity. However, the incentive intensity should not be too high or too low. An extremely low subsidy coefficient has no significant incentive effect on enterprises and universities/institutes, while extremely high subsidy coefficients aggravate the burden of participating green technology innovation on the government. An appropriate subsidy coefficient can strengthen the cooperative relationship among the government, enterprise and university/institute in the pilot project, but it is difficult to realize the comprehensive promotion of government–industry–university–institute collaborative innovation mode through modulating the government subsidy coefficient only.

### 4.4. Influence of Penalty for Breach of Contract W

**Figure 4:**
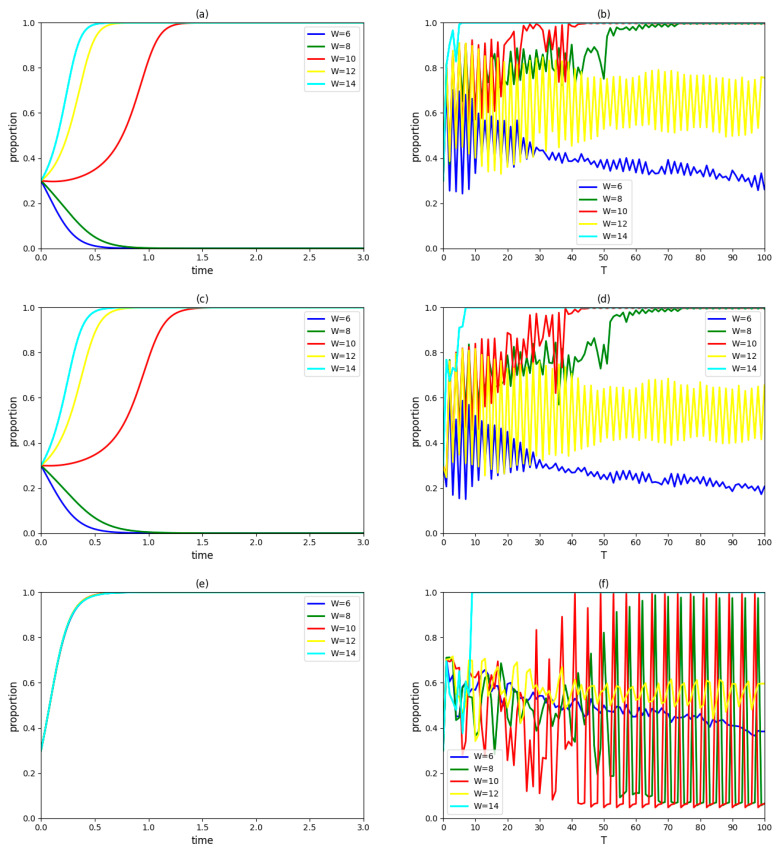
(**a**) The evolutionary paths of an enterprise strategies with different penalties for breaches of contract. (**b**) The proportion of cooperative enterprises in the region over time. (**c**) The evolutionary paths of a university/institute strategies with different penalties for breaches of contract. (**d**) The proportion of cooperative universities/institutes in the region over time. (**e**) The evolutionary paths of government strategies with different penalties for breaches of contract. (**f**) The implementation of the proportion of the government adopting a participation strategy in the region over time.

Assume W takes 6, 8, 10, 12 and 14, respectively, and keep other variables unchanged. The dynamic evolution process of strategic choices of the government, an enterprise and a university in the innovation system and the proportions of those three types of innovation agents accepting the collaboration innovation mode in the region over time are observed as Figure 4.

In Figure 4a,c,e, when the penalty for breach of contract W decreases to 6 or 8, both enterprises and universities/institutes in the innovation system will eventually adopt a non-cooperation strategy; moreover, they will reach a stable state faster under the former condition. When the penalty for breach of contract W increases to 12 or 14, both enterprises and universities/institutes in the innovation system will eventually adopt a cooperation strategy; furthermore, they will reach a stable state faster under the latter condition. The government’s final decision and the rate at which a stable state is achieved are not affected by the penalty for breach of contract W. In terms of the collaborative innovation mode of green technology with government participation and cooperation between an enterprise and a university/institute, the changing penalty for breaches of contract directly affects the profits of the enterprise and the university/institute, which then has an impact on their cooperation intentions [61]; however, it does not affect the government’s profit. Therefore, under the premise of the government being inclined to participate in green technology innovation, increasing the penalty for breaches of contract is conductive to promoting the building of the pilot of government–industry–university–institute collaborative innovation; however, reducing this revenue hinders completion of the pilot.

According to Figure 4b,d,f, the proportions of the government, enterprises and universities/institutes adopting cooperation strategy are continued volatile when W = 6, 8, 10 or 12, which indicates that above penalty schemes cannot make all enterprises and universities/institutes adopt a cooperation strategy; even the full implementation of a participation strategy by the government is unlikely to be realized in the region as well. When W=14, the proportions of the government, enterprises and universities/institutes that accept collaborative innovation in the region will gradually increase to 100%. Only if the penalty for breach of contract exceeds a certain threshold, the government–industry–university–institute collaborative innovation mode will be comprehensive promoted in the region; otherwise, the influence on the willingness of innovation agents will be non-directional.

The penalty for breach of contract is the guarantee of cooperation between the enterprise and university/institute, aiming to avoid the free-riding behavior of their partner in the process of green technology collaborative innovation. For a green technology innovation system formed by an enterprise, a university/institute and the government, the higher the penalty for breach of contract is, the more stable the cooperative relationship among them will be. However, this measure ensuring stable cooperation among the government, enterprises and universities/institutes in the pilot project will not necessarily lead to the comprehensive promotion of the government–industry–university–institute collaborative innovation mode. In order to ensure that all innovation agents in the region actively adopt the green technology collaborative innovation mode, a great demand should be placed on increasing the penalty for breaches of contract.

### 4.5. Influence of Government Penalty K

**Figure 5:**
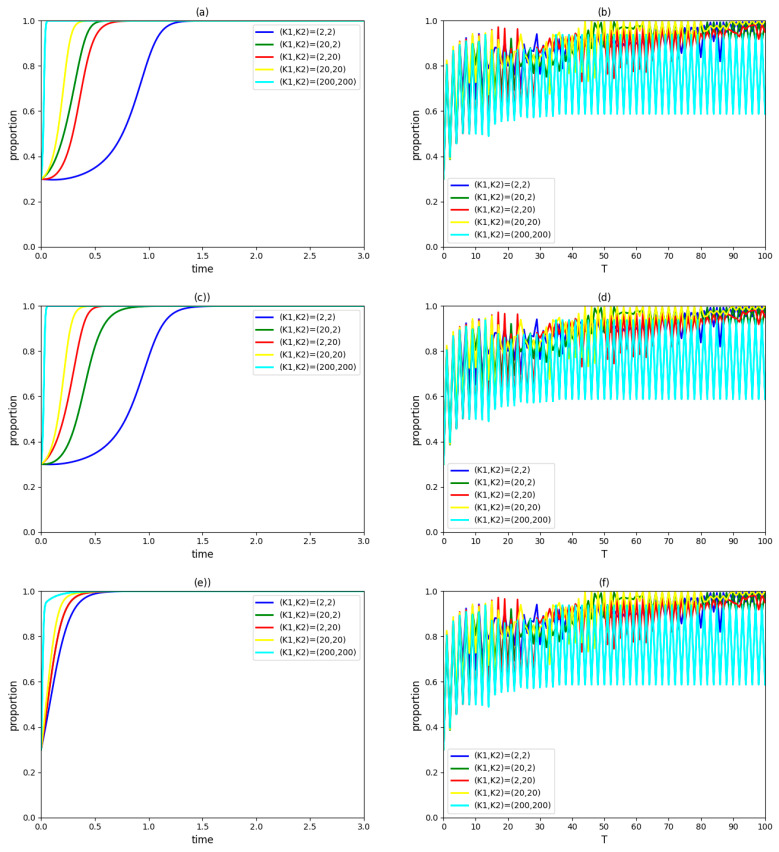
(**a**) The evolutionary paths of an enterprise strategies with different government penalties. (**b**) The proportion of cooperative enterprises in the region over time. (**c**) The evolutionary paths of university/institute strategies with different government penalties. (**d**) The proportion of cooperative universities/institutes in the region over time. (**e**) The evolutionary paths of government strategies with different government penalties. (**f**) The implementation of the proportion of the government adopting a participation strategy in the region over time.

Assume $\left(K_{1},\;K_{2}\right)$ takes (2, 2), (20,2), (2, 20), (20, 20) and (200,200), respectively, and keep other variables unchanged. The dynamic evolution process of the strategic choices of the government, an enterprise and a university in the innovation system and the proportions of those three types of innovation agents accepting the collaboration innovation mode in the region over time are observed as Figure 5.

According to Figure 5a,c,e, under different government penalty schemes, enterprises and universities/institutes in the innovation system will eventually adopt a cooperation strategy, and the government will choose participation strategy. Thereinto, the rates of enterprise and university/institute at which a stable state is achieved when $\left(K_{1},\;K_{2}\right)=\left(20,2\right)$ or (2, 20) are faster than the rates of them when $\left(K_{1},\;K_{2}\right)=\left(2,2\right)$. It shows that, under a specific system, unilaterally increasing the government penalty amount for enterprises or universities/institutes which encroach upon a partner’s profit can not only shorten times taken for enterprises and universities/institutes to reach the stable state, but also have a positive effect on the rate at which a stable state is achieved for the government. When both $K_{1}$ and $K_{2}$ increase to 20, the times taken for the government, enterprise and university/institute to reach the stable state will be further shortened.

In Figure 5b,d,f, when $\left(K_{1},\;K_{2}\right)=\left(2,2\right)$, (20,2), (2, 20) or (20,20), the proportions of the government, enterprises and universities/institutes that accept collaborative innovation in the region cannot increase to 100%. This result remained unchanged, even if the initial values of government penalties $K_{1}$ and $K_{2}$ are expanded 100-fold. Therefore, for a green technology collaborative innovation mode which is guided by the government and with the participation of enterprises and universities/institutes, the implementation effects of a high penalty for breaches of contract and high government penalties are not the same. It indicates that comprehensively promoting the government–industry–university–institute collaborative innovation mode only through increasing the government penalty is difficult. The government’s limited participation in green technology innovation is the reason for this phenomenon. In fact, a high penalty for breach of contract reflects enterprises and universities/institutes preferring collaborative innovation, while the government penalty indicates that the collaborative behaviors of enterprises and universities/institutes deriving from government restrictions [62]. In the stage of pilot project promotion, it is difficult for the government to compel the above two types of innovation agents to strictly adopt a cooperation strategy when acting as a guide.

In general, in order to ensure stable cooperation among the government, enterprises and universities/institutes in the pilot project at the earlier stage, the government penalty should be raised appropriately; in order to successfully promote the pilot project at the later stage, optimizing the method of government participation in green technology collaborative innovation and increasing the revenue of cooperation innovation, the fairness of cooperative innovation revenue distribution, the penalty for breaches of contract, and so on are effective.

## 5. Conclusions

Based on the evolutionary game theory and cellular automata theory, this paper constructs a tripartite evolutionary game model and cellular automata model to study the mechanism of government–industry–university–institute collaborative innovation in green technology from four aspects, including the cost–benefit factor, equitable distribution factor, external incentive factor and penalty factor. The paper has also analyzed how the related factors affect the green technology innovation behaviors of innovation agents via numerical simulation. The results reveal that: (1) Measures ensuring stable cooperation among the government, enterprise and university/institute in a pilot project will not necessarily lead to the comprehensive promotion of the government–industry–university–institute collaborative innovation mode; (2) increasing the revenue of cooperative innovation or improving the fairness of this revenue distribution can make cooperation behaviors of innovation agents emerge and guide an increasing number of innovation agents in the region to accept the collaborative innovation mode; (3) a moderate increase in government subsidies is conductive to the consolidation of the cooperative relationship among the government, enterprises and universities/institutes; however, this measure cannot guide all innovation agents in the region to accept the collaboration innovation mode; (4) a high penalty for breaches of contract can consolidate the cooperative relationship among the government, enterprises and universities/institutes, achieving the goal of guiding all innovation agents in the region to accept the collaboration innovation mode; (5) without a change in the penalty amount for breaches of contract, it is difficult to comprehensively promote the government–industry–university–institute collaborative innovation mode through increasing the government penalty only.

According to the simulation results, the conclusions are obtained as follows: (1) although a successful pilot project plays a demonstrative role regionwide, it does not mean that regional promotion schemes of the government–industry–university–institute collaborative innovation mode can completely copy the pilot project construction scheme, because it is necessary not only to consider the game among different types of innovation agents, but also attach importance to the interaction among the same type of innovation agents in the process of the pilot project promotion; (2) the government, enterprises and universities/institutes are profit-oriented. Increasing the revenue of cooperative innovation can stimulate the willingness of enterprises and universities/institutes to cooperate; meanwhile, a fair distribution of this revenue scheme can enhance the motivation of innovation agents to create and have no impact on the profit of the government as well; (3) although increasing the government subsidy facilitates cooperation between enterprises and universities/institutes, it reduces the profit of the government. Therefore, the government, to a certain extent, can accept to participate in industry–university/institute collaborative innovation in green technology in a subsidy manner, but it is difficult for it to comprehensively implement this green technology collaborative innovation mode in the region; (4) a high penalty for breaches of contract can strongly constrain the cooperative relationship between enterprises and universities/institutes, avoiding the occurrence of free-riding behaviors; (5) because the government plays a guiding role in the green technology collaborative innovation mode, its binding force on enterprise and university/institute is limited. Therefore, that to strengthen all enterprises and universities to adopt a cooperation strategy and promote the green technology collaborative innovation mode in the region relies on government guidance only is unlikely to achieve an ideal effect.

Based on the above conclusions, some suggestions are put forward as follows: (1) in order to promote the government–industry–university–institute collaborative innovation mode, the regional promotion plan should be flexibly formulated and the construction plan of the pilot project should be referenced. Strengthening the social relationship data of enterprises and universities/institutes collection and analysis is also very important, because it can cause the formulated rule of cellular automata to become closer to reality and then accurately predict the impact of specific implementation plans on the diffusion of green technology innovation behavior; (2) the matching degree of the resources of enterprise and university, and the revenue of cooperative innovation should be improved through deepening the cooperative relationship between them. Follow the principle of distribution according to work to make the revenue of cooperative innovation distribution is relatively fair. The measures mentioned above can promote the mode of government–industry–university–institute collaborative innovation in green technology and guide an increasing number of innovation agents in the region to actively adopt cooperation strategy; (3) under the premise of the government being inclined to participate in green technology innovation, it is hard to make all innovation agents in the region work towards a same goal merely depending on government subsidies. Therefore, optimizing the way of government participation and improving the incentive mechanism of government are necessary; (4) in order to avoid free-riding behavior, the penalty for breaches of contract should be set much higher; (5) in order to ensure stable cooperation among the government, enterprises and universities/institutes in a pilot project at the earlier stage, the government penalty should be raised appropriately; in order to successfully promote the pilot project at the later stage, optimizing the way of government participation or formulating a plan to take multiple measures at the same time should be considered.

Although the study proposed a prediction method based on mathematical models and a computer learning algorithm, which can offer help for operating broader cases of government–industry–university–institute green technology collaborative innovation, there are still some limitations: (1) governments participating in green technology collaborative innovation in the form of loans, tax exemptions and so on are not fully considered; (2) the influence of heterogeneities among the same type of enterprises or universities/institutes on the diffusion of green technology innovation behavior is not fully considered; (3) in order to make the economic relationship among enterprises, universities and institutes into a spatial entity, the region constructed in the study is relatively ideal (although the shape of the actual region is different from the ideal region’s, its evolution process and result will not change fundamentally because diffusion mechanisms of innovation behavior are the same under these two conditions.). Based on this study, optimizing the long-term cooperation mode of government–industry–university–institute through exploring the mechanism of different collaborative innovation modes, and analyzing the factors that affect the cooperative behaviors of innovation agents in a complex network and comprehensively promoting the benign collaborative innovation modes will become the next research directions in the future.

## Figures and Tables

**Table 1:** Green technology collaborative innovation game payoff matrix of an enterprise and a university/institute with government participation.

		University/Institute	
		Cooperation	Non-Cooperation
Enterprise	Cooperation	$R_{1}+\alpha \Delta R-\left(1-t_{1}\right)\left(C_{1}{+V}_{1}\right)$, $R_{2}+\beta \Delta R-\left(1-t_{2}\right)\left(C_{2}{+V}_{2}\right)$, $-V_{3}-t_{1}\left(C_{1}{+V}_{1}\right)-t_{2}\left(C_{2}{+V}_{2}\right)$	$R_{1}-\left(1-t_{1}\right)\left(C_{1}{+V}_{1}\right)+W$, $R_{2}-V_{2}{+I}_{2}-W-K_{2}$, $-V_{3}-t_{1}\left(C_{1}{+V}_{1}\right){+K}_{2}$
	Non- cooperation	$R_{1}-V_{1}+I_{1}-W-K_{1}$, $R_{2}-\left(1-t_{2}\right)\left(C_{2}{+V}_{2}\right)+W$, $-V_{3}-t_{2}\left(C_{2}{+V}_{2}\right){+K}_{1}$	$R_{1}-V_{1}{+I}_{1}$, $R_{2}-V_{2}{+I}_{2}$, $-V_{3}$

**Table 2:** Green technology innovation game payoff matrix of an enterprise and a university/institute without government participation.

		University/Institute	
		Cooperation	Non-Cooperation
Enterprise	Cooperation	$R_{1}+\alpha \Delta R-C_{1}-V_{1}$, $R_{2}+\beta \Delta R-C_{2}-V_{2}$, −Q	$R_{1}-C_{1}-V_{1}+W$, $R_{2}-V_{2}+I_{2}-W$, −Q
	Non- cooperation	$R_{1}-V_{1}{+I}_{1}-W$, $R_{2}-C_{2}-V_{2}+W$, −Q	$R_{1}-V_{1}{+I}_{1}$, $R_{2}-V_{2}+I_{2}$, −Q

**Table 3:** Eigenvalues of the Jacobian matrix *J*.

Equilibrium Point	Eigenvalue $\lambda_{1}$	Eigenvalue $\lambda_{2}$	Eigenvalue $\lambda_{3}$
$E_{1}\left(0,\;0,\;0\right)$	$-C_{1}+W-I_{1}$	$-C_{2}+W-I_{2}$	$-V_{3}+Q$
$E_{2}\left(0,\;0,\;1\right)$	$t_{1}\left(C_{1}+V_{1}\right)-C_{1}+W-I_{1}$	$t_{2}\left(C_{2}+V_{2}\right)-C_{2}+W-I_{2}$	$V_{3}-Q$
$E_{3}\left(0,\;1,\;0\right)$	$\alpha \Delta R-C_{1}+W-I_{1}$	$C_{2}-W+I_{2}$	$K_{1}-t_{2}\left(C_{2}+V_{2}\right)-V_{3}+Q$
$E_{4}\left(1,\;0,\;0\right)$	$C_{1}-W+I_{1}$	$\beta \Delta R-C_{2}+W-I_{2}$	$K_{2}-t_{1}\left(C_{1}+V_{1}\right)-V_{3}+Q$
$E_{5}\left(0,\;1,\;1\right)$	$K_{1}+\alpha \Delta R+t_{1}\left(C_{1}+V_{1}\right)-C_{1}+W-I_{1}$	$-t_{2}\left(C_{2}+V_{2}\right)+C_{2}-W+I_{2}$	$t_{2}\left(C_{2}+V_{2}\right)-K_{1}+V_{3}-Q$
$E_{6}\left(1,\;0,\;1\right)$	$-t_{1}\left(C_{1}+V_{1}\right)+C_{1}-W+I_{1}$	$K_{2}+\beta \Delta R+t_{2}\left(C_{2}+V_{2}\right)-C_{2}+W-I_{2}$	$t_{1}\left(C_{1}+V_{1}\right)-K_{2}+V_{3}-Q$
$E_{7}\left(1,\;1,\;0\right)$	$-\alpha \Delta R+C_{1}-W+I_{1}$	$-\beta \Delta R+C_{2}-W+I_{2}$	$-t_{2}\left(C_{2}+V_{2}\right)-t_{1}\left(C_{1}+V_{1}\right)-V_{3}+Q$
$E_{8}\left(1,\;1,\;1\right)$	$-K_{1}-\alpha \Delta R-t_{1}\left(C_{1}+V_{1}\right)+C_{1}-W+I_{1}$	$-K_{2}-\beta \Delta R-t_{2}\left(C_{2}+V_{2}\right)+C_{2}-W+I_{2}$	$t_{2}\left(C_{2}+V_{2}\right)+t_{1}\left(C_{1}+V_{1}\right)+V_{3}-Q$

**Table 4:** Stability analysis of equilibrium points.

Equilibrium Point	Scenario 1				Scenario 2				Scenario 3			
	$\lambda_{1}$	$\lambda_{2}$	$\lambda_{3}$	Equilibrium Results	$\lambda_{1}$	$\lambda_{2}$	$\lambda_{3}$	Equilibrium Results	$\lambda_{1}$	$\lambda_{2}$	$\lambda_{3}$	Equilibrium Results
$E_{1}\left(0,\;0,\;0\right)$	−	−	+	Unstable	+	+	+	Saddle	−	−	+	Unstable
$E_{2}\left(0,\;0,\;1\right)$	−	−	−	ESS	+	+	−	Unstable	+	+	−	Unstable
$E_{3}\left(0,\;1,\;0\right)$	+	+	+/−	Saddle	+	−	+/−	Unstable	+	+	+/−	Saddle
$E_{4}\left(1,\;0,\;0\right)$	+	+	+/−	Saddle	−	+	+/−	Unstable	+	+	+/−	Saddle
$E_{5}\left(0,\;1,\;1\right)$	+	+	+/−	Saddle	+	−	+/−	Unstable	+	−	+/−	Unstable
$E_{6}\left(1,\;0,\;1\right)$	+	+	+/−	Saddle	−	+	+/−	Unstable	−	+	+/−	Unstable
$E_{7}\left(1,\;1,\;0\right)$	−	−	+	Unstable	−	−	+	Unstable	−	−	+	Unstable
$E_{8}\left(1,\;1,\;1\right)$	−	−	−	ESS	−	−	−	ESS	−	−	−	ESS

**Table 5:** Initial values of Coefficients.

**Coefficients**	$V_{1}$ $,\;V_{2}$ $,\;V_{3}$	$R_{1}$ $,\;R_{2}$	ΔR	α, β	$C_{1}$ $,\;C_{2}$	W
Value	5, 2, 10	7, 6	30	0.6, 0.4	4, 4	10
**Coefficients**	$I_{1}$ $,\;I_{2}$	$t_{1}$ $,\;t_{2}$	$K_{1}$ $,\;K_{2}$	Q	a, b, c	T
Value	12, 10	0.05, 0.05	2, 2	20	0.3, 0.3, 0.3	100 ^a^

Note: ^a^ It was proven that the evolution process of green technology innovation behavior diffusion can be shown completely when iteration number T=100.
